# Alleviation of Metabolic Disturbance by Substituting Kanjang High in *Bacillus* for Salt through Modulation of Gut Microbiota in Estrogen-Deficient Rats

**DOI:** 10.3390/foods11131951

**Published:** 2022-06-30

**Authors:** Sunmin Park, Ting Zhang, Yu Yue, Su-Ji Jeong, Myeong-Seon Ryu, Xuangao Wu, Hee-Jong Yang, Do-Yeon Jeong

**Affiliations:** 1 Department of Food and Nutrition, Obesity/Diabetes Research Center, Hoseo University, Asan 31499, Korea; yuyue6491@gmail.com (Y.Y.); yo217@naver.com (S.-J.J.); 2 Department of Bioconvergence, Hoseo University, Asan 31499, Korea; zhangting92925@gmail.com (T.Z.); niyani0@naver.com (X.W.); 3 Department of R & D, Sunchang Research Center for Fermentation Microbes, Sunchang-Gun 56048, Korea; rms6223@naver.com (M.-S.R.); godfiltss@naver.com (H.-J.Y.)

**Keywords:** soy sauce, estrogen deficiency, *Bacillus*, biogenic amines, gut microbiota, metagenome function

## Abstract

A high salt intake may exacerbate menopausal symptoms and substituting for different types of traditionally made kanjang (TMK; soy sauce) may prevent it. This study examined whether substituting salt with lyophilized TMK containing low and high *Bacillus* and biogenic amines in a high-fat diet might modulate the menopausal symptoms and the energy, glucose, and lipid metabolism in ovariectomized (OVX) rats. They were categorized into salt (Control), TMK with high *Bacillus* and low biogenic amines (HBLB), TMK with high *Bacillus* and high biogenic amines (HBHB), TMK with low *Bacillus* and low biogenic amines (LBLB), and TMK with low *Bacillus* and high biogenic amines (LBHB). Sham-operated rats consumed the same diet as the Control. HBLB, HBHB, and LBHB prevented increased tail skin temperature compared to the Control. HBHB and HBLB partially inhibited the increased weight gain and abdominal fat mass by reducing the food efficiency without changing the serum 17β-estradiol concentrations. Serum glucose and insulin concentrations and the insulin resistance index by the homeostatic model assessment for insulin resistance showed a positive association for weight gain. HBLB and HBHB decreased the serum malondialdehyde and tumor-necrosis factor-α levels. Hepatic triglyceride storage was lower in all TMK groups than in the Control, while hepatic glycogen accumulation was higher in the HBLB, HBHB, and LBHB groups than in the Control and LBLB groups. Accordingly, the mRNA expression of peroxisome proliferator-activated receptors-γ(PPAR-γ) was higher in the HBLB and HBHB groups compared to the Control, and that of fatty acid synthase was opposite to PPAR-γ expression. However, HBLB and HBHB improved dyslipidemia and insulin resistance compared to the Control, but their improvement did not reach that of the Normal-control. The acetic acid concentrations in the portal vein were lower in the LBLB than in the Control, while the butyric acid contents were higher in the LBHB and HBLB groups than in the Control. HBHB, HBLB, and LBHB elevated *Akkermansia* and *Lactobacillus*, and HBLB and LBLB increased *Bacteroides* and *Ruminococcus* compared to the Control. Polycyclic aromatic hydrocarbon degradation, bile acid synthesis, and unsaturated fatty acid biosynthesis were significantly higher in the HBLB group than in the Control group. In conclusion, substituting salts to TMK with a high Bacillus content regardless of the bioamine contents partially improved the menopausal symptoms and metabolic disturbance in estrogen-deficient animals.

## 1. Introduction

Obesity is an epidemic worldwide, and its incidence is rising, particularly in menopausal women. An estrogen deficiency induces a hormonal imbalance, disturbing the energy balance and fat relocation, increasing the visceral fat contents, and decreasing lean body mass [1]. It also induces hot flushes, insomnia, depression, and night sweating to reduce the quality of life. Hormonal replacement therapy can protect against menopausal symptoms and metabolic diseases, particularly osteoporosis. It activates the estrogen receptors (ER), both ER-α and ER-β. ER-α activation induces breast and uterine proliferation to elevate the susceptibility to endometrial and breast cancer [2]. Therefore, hormonal replacement therapy is not generally recommended to alleviate menopausal symptoms, and alternative medicine is often used to alleviate menopausal symptoms [1,2].

Chinese medicine is the leading alternative therapy to activate ER-β by inactivating or inhibiting ER-α activation, called selective estrogen receptor modulators [2]. While they activate ER-β and inhibit ER-α activation, they can prevent osteoporosis and bone fracture without breast and uterine proliferation in menopausal women [2]. Thus, herbal extracts having the activity of selective estrogen receptor modulators have been explored to modulate menopausal symptoms, including osteoporosis. Previous studies have revealed that several herbal extracts, such as soybeans, ginseng, Dong Quai, *Tetragonia tetragonioides* (Pall.) Kuntze, *Opuntia ficus-indica* Mill, *Dioscorea nipponica* Makino, black cohosh, and others have alleviated menopausal symptoms [2,3].

Isoflavones in soybeans are estrogen analogs to activate ER-α and ER-β, and their intake reduces menopausal symptoms. Randomized clinical trials have shown that the intake of 1–2 soy servings per day (20–80 mg isoflavones) improves menopausal symptoms, especially vasomotor symptoms [4,5]. However, there is some controversy about whether soy intake may induce breast cancer, even though observational studies in American and Asian populations have shown that regularly consuming soy foods has a lower risk for breast cancer [6,7]. Isoflavonoids in fermented soybeans, including traditionally made kanjang (TMK) are better substrates to produce equol (the potent estrogen activity) from daidzein by fecal bacteria [8,9,10]. It is a potent activator of estrogen receptors to promote osteoblast proliferation and differentiation [11]. The intake of fermented soybean with *Bacillus* spp. alleviates menopausal symptoms in menopausal women and ovariectomized (OVX) rats. The 24-week intervention of *Bacillus subtilus* is shown to improve bone mineral density (BMD) by inhibiting bone resorption and increasing the relative abundance of *Bifidobacterium* in gut microbiota in healthy postmenopausal women [12]. Therefore, TMK potentially enhances the menopausal symptoms by fermented isoflavones and fibers, *Bacillus* spp., and postbiotics in postmenopausal women.

A high sodium intake (>2 g/day) was reported to lower the bone mineral density by 28% and worsen the overall mood than low sodium intake in postmenopausal women [13,14]. Asians have a higher sodium intake than Caucasians because the staple food is rice and soup. Most fermented soybeans contain salts and are used as a seasoning dish. The beneficial health effects of fermented soybean products may be counteracted with the contents of sodium, aflatoxin, and biogenic amines [15,16]. However, a few studies have shown that TMK, a fermented soy sauce, is beneficial for energy, glucose, lipid, and bone metabolism in menopausal women when the sodium contents are equivalent [17,18]. Instead of regular salts, TMK intake, including isoflavonoids and *Bacillus* spp., may prevent menopausal symptoms and bone mineral density loss, compared to regular salt intake, when the sodium contents are equivalent. This study tested the hypothesis that different types of TMK containing low and high levels of biogenic amines and *Bacillus* spp. might influence menopausal symptoms and bone mineral density differently. This hypothesis was examined in OVX rats fed a high-fat diet containing different types of TMK or regular salt, and their action mechanisms were explored.

## 2. Materials and Methods

### 2.1. General Production Process of TMK and Its Collection

TMK is generally produced using a two-step process: Meju was made by fermenting crushed boiled soybeans with rice straw at approximately 20–25 °C for 40–50 days. Meju was further fermented in 16–18 Brix salty water for 40–60 days, and then the solution and remaining residues were separated, called TMK and traditionally made doenjang, respectively. Several TMK samples were purchased from different provinces in Korea. In a preliminary study, the contents of sodium, beneficial and harmful bacteria, and biogenic amines were measured. Four TMK products were selected for the animal study to determine if they alleviated the menopausal symptoms. The selection criteria were low and high in beneficial bacteria and biogenic amines. Four different types of kanjang were selected: high *Bacillus* spp. + low biogenic amine, high *Bacillus* spp. + high biogenic amine, low *Bacillus* spp. + low biogenic amine, and low *Bacillus* spp. + high biogenic amine in our preliminary screening study. 

According to the Korea Food and Drug Administration guidelines, sodium and aflatoxin contents in various TMK were measured. The sodium contents in the TMK were assessed using inductively coupled plasma atomic emission spectroscopy (ICP-AES, Thermo IRIS Intrepid II XDL, Waltham, MA, USA) after proteins in TMK were digested with nitric acid. The oxidant and acetylene flows were 10.0 L/min and 2.5 L/min, respectively. The flame type was air acetylene, and the wavelength was 589.0 nm. TMK was mixed with 70% methanol containing 1% NaCl and filtered. The filtrates were placed into the aflatoxin test column to elute aflatoxin. The aflatoxin contents in the eluents were determined using high-performance liquid chromatography (HPLC), attached with Shiseido UG 120 (4.6 × 250 mm, 5 μm, Osaka, Japan) column and fluorescence detector (excitation: 360 nm, measurement 450 nm; Agilent 1200 series, Agilent Technologies, Santa Clara, CA, USA). The mobile phase was made of mixing water and acetonitrile (75:25, *v*/*v*; JT baker, Phillipsburg, NJ, USA); the injection volume and flow rate were 10 μL and 1.0 mL/min, respectively.

The contents of histamine and tyramine as biogenic amines were determined as described previously [19]. TMK was mixed with 0.1 g/L of 1,7-diaminoheptane as an internal standard and added 1% dansyl chloride (Sigma–Aldrich, St. Louise, MO, USA) and a saturated Na_2_CO_3_ solution to make derivatives. Diethyl ether (Samchun, Seoul, Korea) was added to the solution and mixed for 3 min, and the supernatants were separated. Their solvent was removed, and the residues were dissolved in acetonitrile (Duksan, Seoul, Korea). The 0.1–100 mg/L histamine and tyramine as standards were prepared in a 0.01 N HCl solution. Its biogenic amine contents were then measured by HPLC using a Cepcell Pak C18 column (2.0 × 250 mm) [19].

The proportion of beneficial and pathogenic bacteria was calculated from the bacteria contents measured using the next-generation sequencing procedure. TMK and fecal DNA were extracted and were preprocessed for assessing the sequences measured by the Illumina MiSeq standard operating procedure on a Genome Sequencer FLX plus (454 Life Sciences, Branford, CT, USA), performed in Microbial Institute for Fermentation Industry (Soon Chang, Korea). The 16S amplicon sequences of each TMK and fecal bacteria were used to identify the bacteria taxonomy and counts by Mothur program v.1.36 (University of Michigan, Ann Arbor, MI, USA). After removing the operational taxonomic units below 10,000 reads, the relative bacteria counts of each TMK and feces were evaluated in the taxonomy levels at the family, order, genus, and species.

### 2.2. Ovariectomy Operation

Sixty female eight-week Sprague–Dawley rats (167 ± 10 g) purchased from DBL (Yeumsung-Kun, Korea) were adapted in the animal facility Invivo Company (Nonsan-si, Korea) for one week. Female Sprague–Dawley rats aged eight weeks resided in an individual stainless-steel cage in the animal facility at Hoseo University maintained at 23 ± 2 °C with a 12-h light/dark cycle. This study was conducted according to the National Institute of Health Guide for the Care and Use of Laboratory Animals with the Invivo Company Animal Care and Use Committee (IV-RA-13-2019-35). 

Anesthesia was maintained with the subcutaneous injection of a ketamine/xylazine mixture (100 and 10 mg/kg body weight) during the OVX procedure [20]. Both ovaries were dissected with scissors after ligating the end of each oviduct. The OVX groups included 10 OVX rats per group, and ten rats had a sham operation.

### 2.3. Diet Preparation

All rats had high-fat diets to exacerbate the estrogen-deficient status [20,21,22]. A high-fat diet was generated with a semi-purified method based on the American Institute of Nutrition 93 formulation for experimental animals. The primary sources of carbohydrate, protein, and fat were starch plus sugar, casein, and lard (CJ Co., Seoul, Korea), respectively. The TMK nutrient composition was analyzed, and its carbohydrate, fat, protein, and sodium contents were excluded from the dietary contents. The mineral mixture was made without salts, and TMK or salt was added to the diet. According to the nutrient contents in TMK, lyophilized TMK was added to a 43% fat diet to make equivalent carbohydrate, fat, protein, and sodium contents in all groups. The salt content in each diet was 5.9 g salt/kg diet. Each TMK powder was blended thoroughly into the vitamin and mineral mixture without sodium and sifted to remove the lumps. The vitamin and mineral mixtures were added into the designated amounts of starch, casein, and lard and resifted to make the diet contents homogenous. The primary nutrient compositions were equivalent in each group.

### 2.4. Experimental Design

According to dietary sodium source, fifty OVX rats were randomly divided into the following five groups: they were fed (1) salt (Control), (2) lyophilized TMK with high *Bacillus* and low biogenic amines (HBLB), (3) lyophilized TMK with high *Bacillus* and high biogenic amines (HBHB), (4) lyophilized TMK with low *Bacillus* and low biogenic amines (LBLB), and (5) lyophilized TMK with low *Bacillus* and high biogenic amines (LBHB). The sham-operated rats had the same diet as the Control. All rats in each group consumed the assigned diets for 12 weeks. Body weight and weekly food and water intake were measured at 10 AM every Tuesday.

### 2.5. Tail Skin Temperature

The tail skin temperature as hot flushes in menopausal symptoms was measured three times 10 min each week during the light cycle by an infrared thermometer for small rodents (BIO-152-IRB, Bioseb, Chaville, France) [22].

### 2.6. Body Composition

Before and after the intervention, body composition, including BMD, lean body mass, and fat mass, was determined in each rat using a dual-energy X-ray absorptiometer (DEXA) calibrated with a phantom supplied by the manufacturer (Norland Medical Systems Inc., Fort Atkinson, WI, USA). The DEXA instrument was equipped with software to assess the body composition in rodents. After anesthesia with the ketamine and xylazine mixture, a rat was laid down in the prone position with the rear legs held in external rotation and the articulations of the hips, knees, and ankles in 90° flexion. After scanning, the BMD in the lumbar spine and leg, the lean body mass in the hip and leg, and the fat mass in the abdominal and leg fat mass were calculated [22].

### 2.7. Glucose Homeostasis and Sample Collection

After overnight fasting in the 11th week, each rat was orally given 2 g of glucose/kg body weight to conduct the oral glucose tolerance test (OGTT). Every 10 min until 120 min, the serum glucose concentration was measured from the tail blood using a Glucose Analyzer II (Beckman, Palo Alto, CA, USA). The serum insulin concentration was assessed at 0, 20, 40, 90, and 120 min using a radioimmunoassay kit (Linco Research, Billerica, MA, USA). The total area under the curve (AUC) for the serum glucose and insulin concentrations from the OGTT were calculated using the trapezoidal rule. At three days post-OGTT, each rat had 6 h fasting and had an intraperitoneal injection of insulin (0.75 U/kg body weight) to perform an intraperitoneal insulin tolerance test (IPITT) to assess insulin sensitivity. The serum glucose concentrations were measured every 15 min for 90 min using a Glucose Analyzer II (Beckman-Coulter, Palo Alto, CA, USA).

Two days post-IPITT, the body composition was estimated using DEXA in the anesthetized rats. The uteri, epididymal and retroperitoneal fat were excised and weighed. The uterus index was calculated with the equation of the uterus weight divided by body weight. The serum was separated by centrifuging the blood collected from the heart at 3000 rpm for 20 min. The serum and tissues were then stored at −70 °C for future use.

The homeostasis model assessment estimate for assessing insulin resistance (HOMA-IR) was estimated by multiplying fasting insulin (µIU/mL) and fasting glucose (mM) divided by 22.5. The serum 17β-estradiol levels were measured using enzyme-linked immunosorbent assay (ELISA) kits (Enzo Life Sciences, NY, USA). The serum concentrations of total cholesterol, HDL cholesterol (HDL-C), and triglycerides were assayed using the respective colorimetry kits (Asan Pharmaceutical, Seoul, Korea). The serum LDL-C concentrations were calculated using the Friedewald equation (LDL-C = total cholesterol − HDL-C − triglyceride/5). The ELISA kits were used to determine serum tumor-necrosis factor (TNF)-α (R & D Systems, Minneapolis, MN, USA), lipid peroxide (malondialdehyde; Abcam, Cambridge, UK), and aldosterone concentrations (Enzo Life Sciences, Farmingdale, NY, USA).

### 2.8. Islet Isolation, Total RNA Generation, and Gene Expression by Real-Time PCR

According to the manufacturer’s method, the liver was homogenized with TRIzol reagent (Gibco-BRL, Rockville, MD, USA), and the total RNA was separated from the aqueous part after centrifugation. Its equal amount in each sample was mixed with Superscript III reverse transcriptase, and a polymerase chain reaction (PCR) was implemented to synthesize cDNA. The synthesized cDNAs were equally added to the SYBR Green mix (Bio-Rad, Richmond, CA) along with the primers for specific genes, and a real-time PCR was conducted in the real-time PCR instrument (Bio-Rad) under optimal conditions for thermal cycling. The expression of the inflammatory cytokine, TNF-α, and interleukin (IL)-1β, were determined using corresponding primers, as previously described [23]. The expression of the genes of interest was normalized to that of the β-actin gene. A cycle of threshold (CT) for each sample was used for quantifying the expression levels of the interested genes by the comparative CT method (ΔΔCT method). ΔCT was calculated as: ΔCT = CT (target gene) − CT (endogenous reference gene, β-actin). The differences in gene expression were calculated as 2^−ΔΔCT^ in ΔΔCT = ΔCT for the treatment group − ΔCT for the control group.

### 2.9. Serum SCFA Concentrations and Gut Microbiome by Next-Generation Sequencing (NGS)

The serum was isolated from the blood collected from the portal vein and mixed with ethanol (Duksan, Daejeon, Korea). 1N HCl was added to the mixture (100:1) and centrifuged at 15,000 rpm and 4 °C for 15 min. SCFA concentrations in the supernatants were assayed by gas chromatography (GC, Clarus 680 GAS, PerkinElmer, Waltham, MA, USA) equipped with an Elite-FFAP 30 m × 0.25 mm × 0.25 μm capillary column. Helium was used as the carrier gas at a flow rate of 1 mL/min [24]. Acetate, propionate, and butyrate (1 mM; Sigma Co., St. Louise, MO, USA) were used as the external standards.

The fecal microbiota communities from the cecum were examined using next-generation sequencing procedures, as described in the previous study [25]. The bacterial DNA from the feces was isolated using a Power Water DNA Isolation Kit (Qiagen, Valencia, CA, USA) and amplified with 16S amplicon primers by PCR. Their libraries were prepared using the PCR products according to the GS FLX plus library prep guide [26]. According to the manufacturer’s instructions, the PCR amplification program was run using the 16S universal primers in the FastStart High Fidelity PCR System (Roche, Basel, Switzerland). The fecal bacterial DNA was sequenced using the Illumina MiSeq standard operating procedure and a Genome Sequencer FLX plus (454 Life Sciences) (Macrogen, Seoul, Korea). 

The 16S amplicon sequences were processed using Mothur v.1.36. according to Miseq’s standard operating procedure in each fecal sample. The sequences were aligned using Silva reference alignment v.12350 (Bremen, Germany), and bacteria counts and taxonomy identifications were determined as described previously [24,26]. The relative bacteria counts were calculated in taxonomic assignment for each sample, and the α- and β-diversity of the fecal bacteria were analyzed. The β-diversity was calculated based on an unweighed UniFraq distance matrix and visualized using the R package. The separation of each group in β-diversity was analyzed using the permutation-based variance analysis (PERMANOVA). Network analysis determined the links among gut bacteria at the genus level, SCFA, visceral fat mass, and glucose metabolism were determined [27].

### 2.10. Metagenome Functions of the Fecal Bacteria by PICRUSt2 Pipeline Analysis

The metabolic functions of gut microbiota were predicted from the fasta files and count tables of fecal bacteria using PICRUSt2 [28]. The metabolic functions were estimated using the Kyoto Encyclopedia of Genes and Genomes (KEGG) Orthologues mapped by the KEGG mapper (https://www.genome.jp/kegg/tool/map_pathway1.html; accessed on 5 February 2022) [29]. The gut microbiome was used to explore the metabolic functions of each rat.

### 2.11. Statistical Analysis

SAS software version 7 (SAS Institute, Cary, NA, USA) was used for statistical analysis. The sample size for each group was determined using a G power program (power = 0.90 and effect size = 0.5), and the calculated sample size was 10 per group. The results are expressed as the means ± standard deviation (SD) if they had a normal distribution in Proc univariate. The measurements were analyzed statistically using one-way ANOVA. The differences among the groups were assessed using a Tukey’s test. The statistical differences were considered significant at *p* < 0.05.

## 3. Results

### 3.1. General Characteristics of TMK with Different Levels of Bacillus and Biogenic Amines

**Figure 1:**
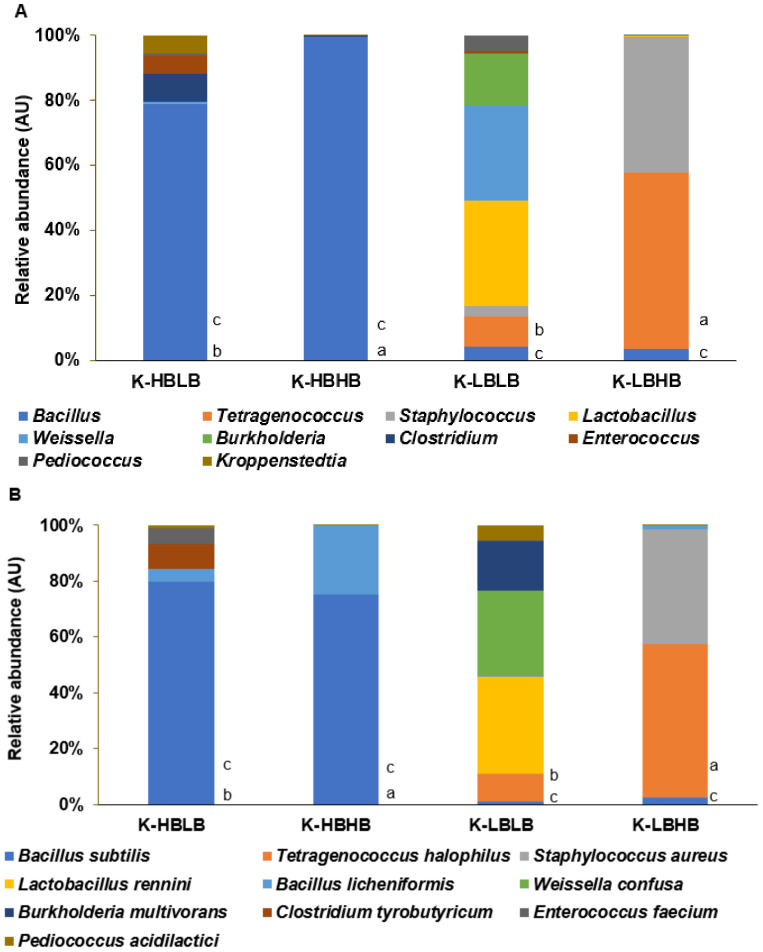
Relative abundance of bacteria in traditionally made kanjang according to Bacillus and bioamines contents. (**A**). The genus level (**B**). The species level. K-HBLB, TMK with high contents of *Bacillus* spp. and high biogenic amines; K-HBHB, TMK with high contents of *Bacillus* spp. and low biogenic amines; K-LBHB, TMK with low contents of *Bacillus* spp. and high biogenic amines; K-LBLB, TMK with low contents of *Bacillus* spp. and low biogenic amines. a–c Different letters beside the bars indicated a significant difference among the groups by Tukey test at *p* < 0.05.

HBHB mainly contained Bacillus subtilus and Bacillus licheniformis, while HBLB included Bacillus subtilus, Bacillus licheniformis, Clostridium tyrobutyricum, Enterococcus faecium, and Pediococcus acidilactici (Figure 1A,B). HBLB contained 79.0% beneficial bacteria and 7.60 mg/kg histamine and 33.3 mg/kg tyramine. HBHB included similar amounts of Bacillus bacteria to HBLB, but the histamine and tyramine contents were 30 times higher than HBLB. However, LBLB and LBHB included very low Bacillus spp. LBHB contained Tetragencoccus and Staphylococcus, while LBLB contained high Lactobacillus, Weissella, and Burkholderia (Figure 1A,B). LBLB had low levels of Bacillus spp. (39.6%) and low levels of biogenic amines (29.6 mg/kg histamine and 155 kg/kg tyramine). LBHB contained low levels of Bacillus spp. (1.94) and high levels of biogenic amines (214 mg/kg histamine and 1009 mg/kg tyramine) (Table 1). The differences in the biogenic amine contents but not Bacillus spp. were associated with the sodium contents. TMK with lower sodium contents (<7.0%) had high biogenic amine contents. No aflatoxin was detected in any TMK selected (Table 1).

### 3.2. Serum 17β-Estradiol Concentration, Uterine Weight, and Tail Skin Temperature

**Figure 2:**
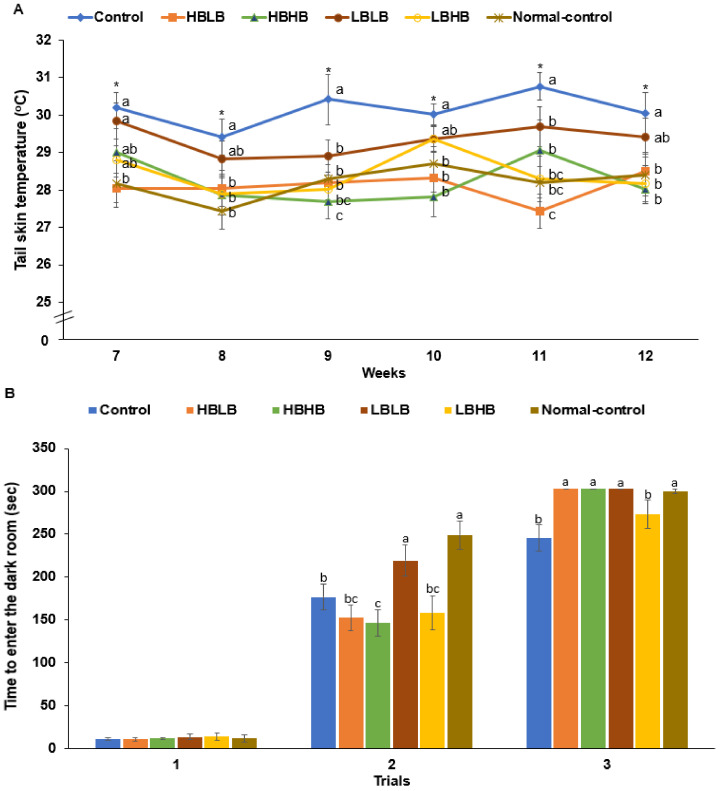
Tail skin temperature and passive avoidance test after ovariectomized rats. (**A**). Tail skin temperature during 7–12 weeks (**B**). Time to enter the darkroom. Dots or bars and error bars represent the means ± standard deviations (*n* = 10). HBLB, the rats fed TMK with high contents of *Bacillus* spp. and high biogenic amines; HBHB, the rats fed TMK with high contents of *Bacillus* spp. and low biogenic amines; LBHB, the rats fed TMK with low contents of *Bacillus* spp. and high biogenic amines; LBLB, the rats fed TMK with low contents of *Bacillus* spp. and low biogenic amines. * Significantly different among the groups at *p* < 0.05. a–c Different letters on the bars indicated a significant difference among the groups by Tukey test at *p* < 0.05.

Uterine weight and serum 17β-estradiol concentrations were 6.6 and 4.7 times lower, respectively, in the Control than in the Normal-control and different types of TMK did not change them from the Control (Table 2). The decrement was involved in disturbed metabolism to develop menopausal symptoms. Serum aldosterone concentrations were also lower in OVX rats than in the Normal-control, whereas all TMK groups increased the concentrations compared to the Control, and HBHB and HBLB elevated more than LBLB (Table 2). The tail skin temperature representing hot flush in menopausal women was elevated in the estrogen-deficient rats compared to the Normal-control rats from the 7th to 12th week (Figure 2A). TMK intake decreased tail skin temperature compared to the Control and HBLB, HBHB, and LBHB decreased it, similar to the Normal-control group (Figure 2A). In the short-term memory test, LBLB showed higher memory function than other groups in the second trial (Figure 2B). In the third trial of a passive avoidant test, the latency time to entering the darkroom was lower in the Control than in Normal-control, and all TMK groups showed an increase in latency. All except for the LBHB group reached the maximum time (Figure 2B), indicating the rats fed TMK improved their short-memory function.

### 3.3. Energy Metabolism and Body Composition

The weight gain during the entire treatment period was much higher in the Control group than in the Normal-control group. Four types of TMK inhibited weight gain in the OVX rats, but the increment in weight gain was not inhibited as much as in the Normal-control group (Table 2). Among the TMK groups, weight gain was prevented the most in the HBHB group. The uterine and retroperitoneal fat mass exhibited a similar trend of weight gain, while the visceral fat contents, summing uterine, and retroperitoneal fat were lowest in the HBHB among all treatment groups (Table 2). On the other hand, there were no significant differences in the food intake among all groups. The weight gain in the OVX rats depended on the food efficiency calculated by dividing the weight gain by food intake × 100. The food efficiency was higher in the Control than in the Normal-control, and all TMK intake decreased food efficiency in the OVX rats. The HBHB group showed the lowest percentage among the OVX rats, but the decrease was smaller than the Normal-control (Table 2).

**Figure 3:**
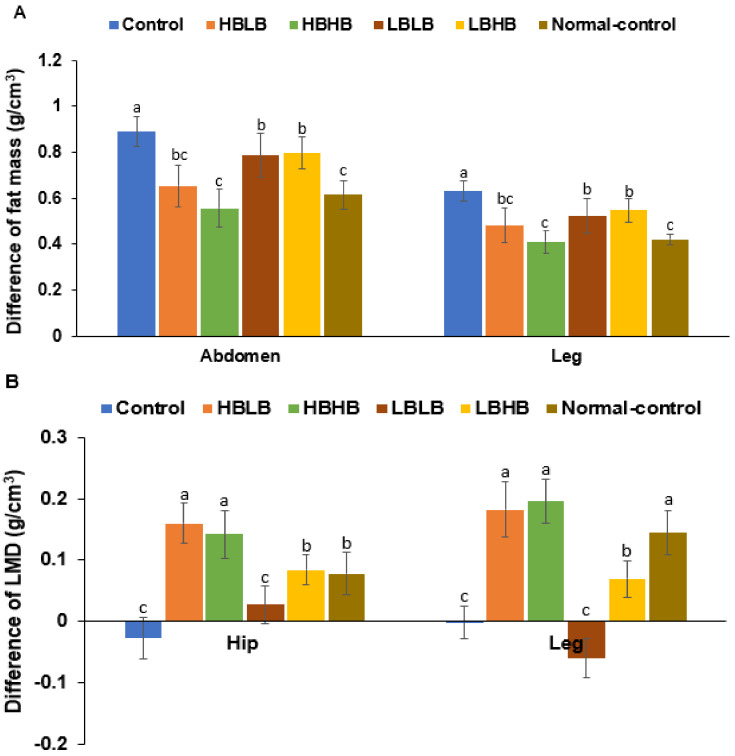

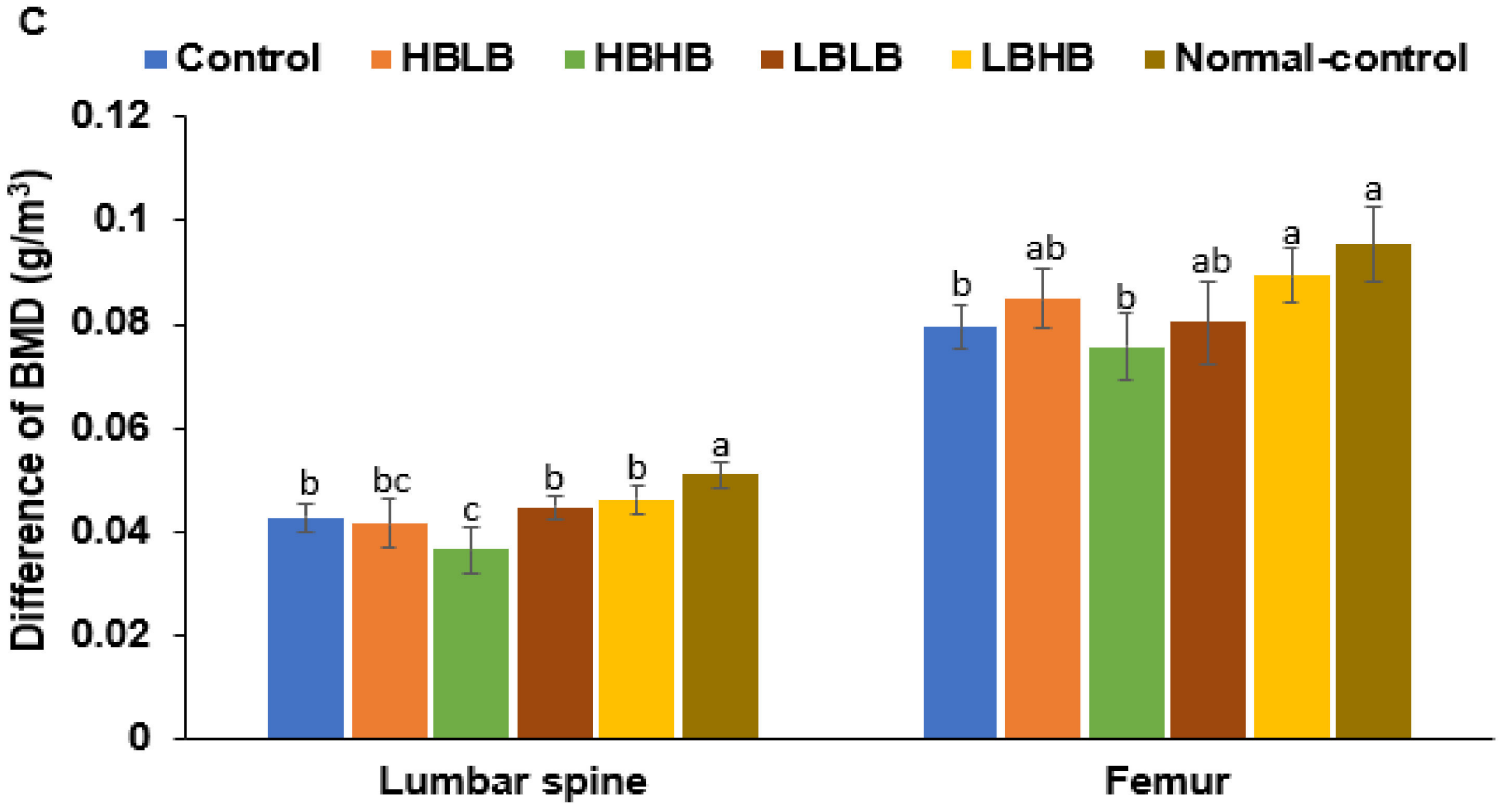
The differences in body composition (**A**). Differences of fat mass in abdomen and leg before and after ovariectomy. (**B**). Differences in lean body mass in the hip and leg before and after ovariectomy. (**C**). Differences in bone mineral density in the lumbar spine and femur before and after ovariectomy. Bars and error bars represent the means ± standard deviations (*n* = 10). HBLB, the rats fed TMK with high contents of *Bacillus* spp. and high biogenic amines; HBHB, the rats fed TMK with high contents of *Bacillus* spp. and low biogenic amines; LBHB, the rats fed TMK with low contents of *Bacillus* spp. and high biogenic amines; LBLB, the rats fed TMK with low contents of *Bacillus* spp. and low biogenic amines. a–c Different letters on the bars indicated a significant difference among the groups by the Tukey test at *p* < 0.05.

Consistent with the excised weight of visceral fat, abdominal fat content measured by DEXA was higher in the Control than the Normal-control while TMK prevented the increase in abdominal fat mass compared to the Control. Among the TMK groups, the HBLB and HBHB groups had a similar abdominal fat mass to the Normal-control (Figure 3A). Fat mass in the leg exhibited the same pattern as the abdominal fat mass; that of the HBLB and HBHB groups was equivalent to the Control (Figure 3A). The lean body mass in the hip and leg did not change in the Control group during the 12-week experiment periods, although it increased in the Normal-control and TMK treatments except for LBLB (Figure 3B). The HBLB and HBHB groups increased the lean body mass in the hip and leg more than the Normal-control group, and the LBHB group had a similar lean body mass to the Normal-control (Figure 3B). The BMD in the lumbar spine and femur increased in all groups during the experimental periods, and its increase was lower in the Control group than in the Normal-control group (Figure 3C). TMK administrations did not alter the BMD in the lumbar spine and femur, but the HBLB and LBHB groups increased femur BMD compared to the Control (Figure 3C). Therefore, OVX disturbed the body composition, and the HBLB and HBHB groups protected against the disturbance of body composition.

### 3.4. Glucose Metabolism

The serum glucose concentrations in overnight-fasted status were much higher in the Control group than in the Normal-control group (Table 3). The HBLB and HBHB groups showed significantly lower concentrations than the Control group, but they did not reduce them to the level of the Normal-control. The serum insulin concentrations were also higher in the Control group than in the Normal-control while they were lower in the HBLB and HBHB groups than in the Control group (Table 3). The HOMA-IR, an insulin resistance index, was much higher in the Control than the Normal-control while the HBLB and HBHB treatments prevented the increase. The LBLB group showed similar levels to the Control (Table 3).

**Figure 4:**
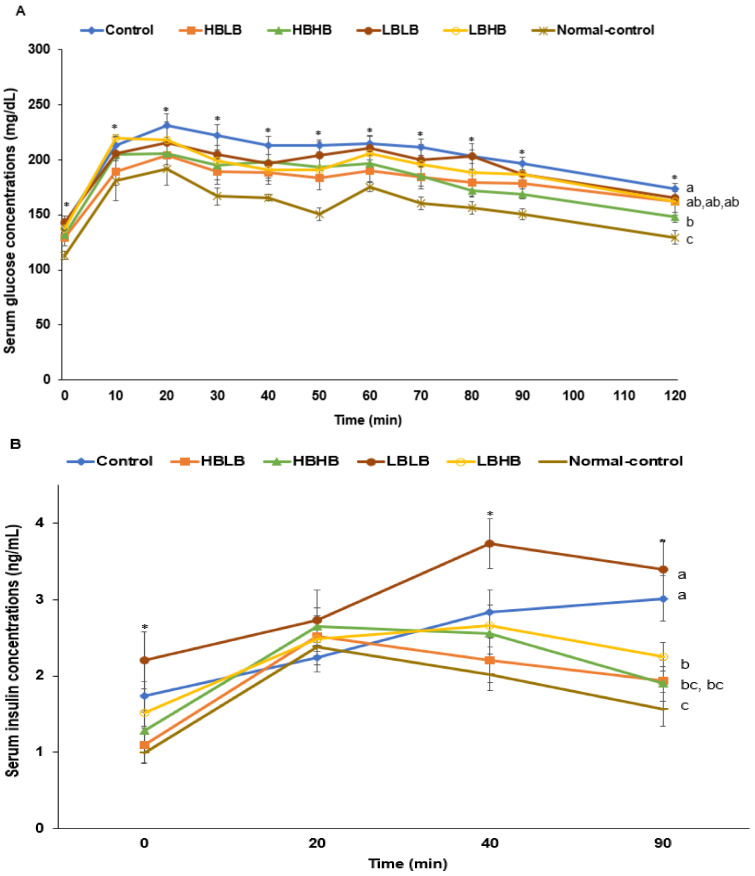

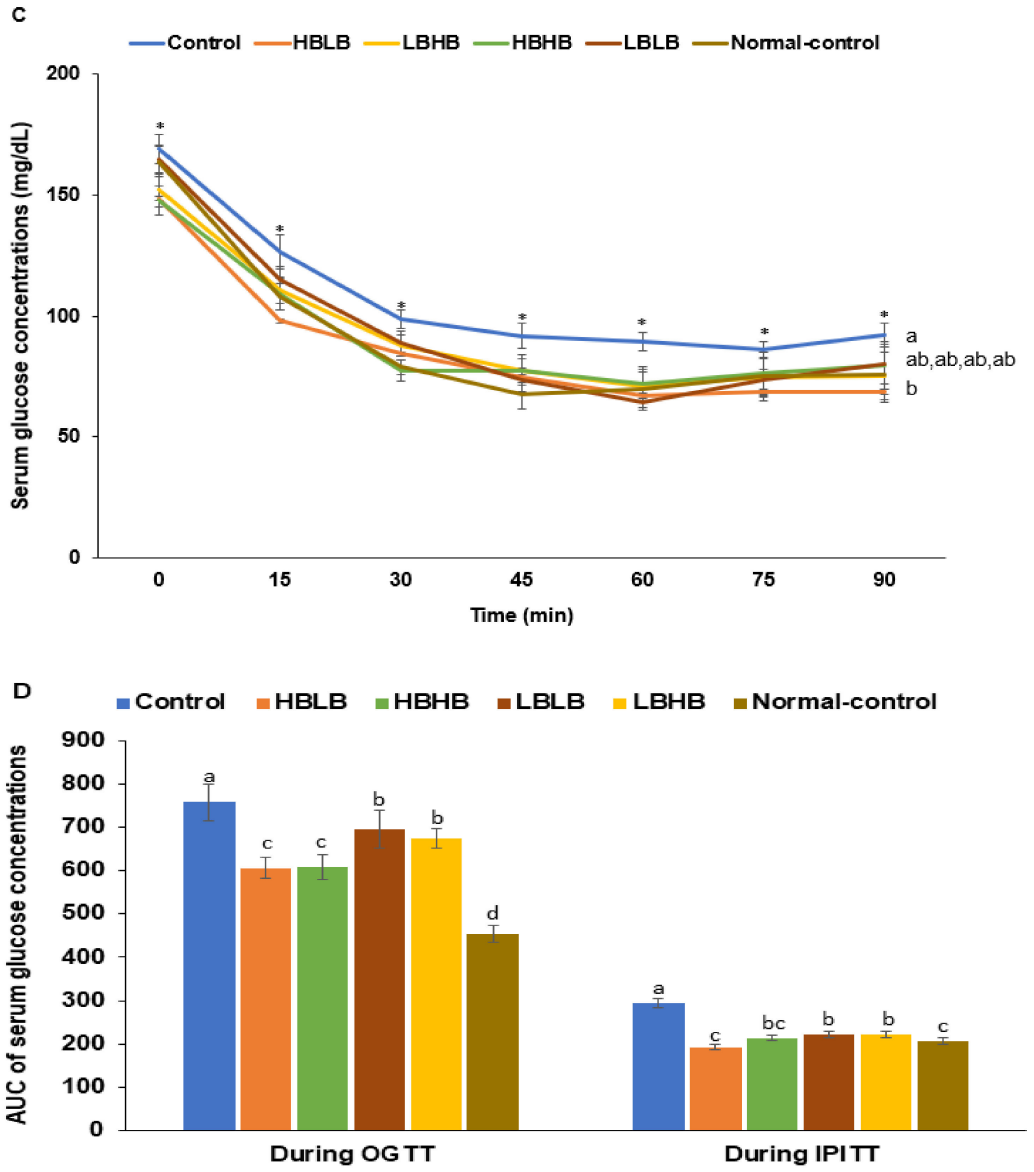
Serum glucose concentrations during oral glucose tolerance (OGTT) and intraperitoneal insulin tolerance test (IPITT). (**A**). Changes in serum glucose concentrations after oral intake of 2 g glucose per kg body weight during OGTT. (**B**). Changes in serum insulin concentration during IPITT. (**C**). Changes in serum glucose concentration after intraperitoneal injection of 1 U insulin per kg body weight. (**D**). The area under the curve of serum glucose concentration during OGTT and IPITT. Dots or bars and error bars represent the means ± standard deviations (*n* = 10). HBLB, the rats fed TMK with high contents of *Bacillus* spp. and high biogenic amines; HBHB, the rats fed TMK with high contents of *Bacillus* spp. and low biogenic amines; LBHB, the rats fed TMK with low contents of *Bacillus* spp. and high biogenic amines; LBLB, the rats fed TMK with low contents of *Bacillus* spp. and low biogenic amines. OGTT, oral glucose tolerance test; IPITT, intraperitoneal insulin tolerance test. * Significantly different among the groups at *p* < 0.05. a–d Different letters on the bars indicated a significant difference among the groups by Tukey test at *p* < 0.05.

During OGTT, the serum glucose concentrations increased until 20 min and decreased in all rats. The peak serum glucose concentrations were highest in the Control group among all groups, and those in the HBLB and HBHB groups were lower than in the Control (Figure 4A). They were lowest in the HBHB group but higher than the Normal-control (Figure 4A). The AUC of serum glucose concentrations during OGTT was higher in the Control than in the Normal-control, while the increase was prevented in the HBLB, HBHB, and LBHB groups compared to the Control (Figure 4B).

During OGTT, serum insulin concentrations increased until 20 min and decreased in most groups except the Control. They increased after 40 min only in the Control group. The serum insulin concentrations at the peak were higher in the Control than in the Normal-control, and they were similar in the HBLB and Normal-control groups (Figure 4C). The LBLB had a higher serum insulin concentration than the Control group (Figure 4C). The serum insulin concentrations at 90 min were higher in the Control and LBLB groups than in the other groups (Figure 4C).

At six-hour fasting, serum glucose concentrations were higher in the Control than in the Normal-control, and they were lower in the HBLB, HBHB, and LBHB groups than in the Control (Figure 4D). During IPITT, the serum glucose concentrations decreased until 30 min and were sustained during 45–90 min in all rats. The four types of TMK intakes decreased the serum glucose concentration after insulin injection compared to the Control and were close to the Normal-control (Figure 4D). The AUC of serum glucose concentrations during IPITT was higher in the Control than the Normal-control while TMK intake lowered it. Among the different TMK types, HBLB lowered the AUC similar to the Normal-control (Figure 4B).

### 3.5. Lipid Profiles

The overall lipid profiles were higher in the Control than the Normal-control, and TMK differently altered them in the OVX rats. The LBLB and LBHB groups showed lower serum total cholesterol concentrations than the other groups (Table 3). The serum HDL concentrations in the Control were the lowest, whereas those in the Normal-control were the highest. Among the TMK types, serum HDL concentrations increased in the order of the LBLB, LBHB, HBHB, and HBLB groups, but those in the HBLB did not increase as much as the Normal-control (Table 3). Serum triglyceride concentrations were much higher in the Control than in the Normal-control and HBHB groups and lower in the LBHB group than in the Control. The serum LDL concentrations estimated using Friedewald’s equation showed similar serum triglyceride concentrations, but all TMK intakes lowered them (Table 3).

The serum TNF-α concentrations, an inflammation index, were higher in the Control than in the Normal-control and lowered in the HBLB, HBHB, and LBHB groups than in the Control. The HBLB group showed lower serum TNF-α levels than the Normal-control (Table 3). As an oxidative stress index, the serum malondialdehyde concentrations were also higher in the Control than in the Normal-control. The HBLB and HBHB groups showed lower serum malondialdehyde concentrations (Table 3).

### 3.6. Liver Metabolism

Glycogen storage in the liver was lower in the Control than in the Normal-control, and this decrease was prevented in the HBLB, HBHB, and LBHB groups (Table 4). By contrast, hepatic triglyceride accumulation was higher in the Control than in the Normal-control. The rats that consumed all TMK showed a decrease, and the HBLB and HBHB groups had similar storage to the Normal-control (Table 4).

As hepatic triglyceride storage is associated with triglyceride synthesis and insulin resistance, the mRNA expression of FAS and peroxisome proliferator-activated receptors (PPAR)-γ was determined (Table 4). FAS mRNA expression was higher in the Control than in the Normal-control, and its increase was prevented in the HBLB, HBHB, and LBHB groups (Table 4). In contrast, PPAR-γ mRNA expression was lower in the Control group than in the Normal-control and higher in the HBLB and HBHB groups. On the other hand, the mRNA expression of SREBP-1c involved in cholesterol synthesis was not significantly different among all groups. The liver damage indexes, serum AST and ALT concentrations were higher in the Control than the Normal-control, and all TMK intakes lowered them (Table 4).

### 3.7. SCFA Concentration from the Portal Vein Blood

**Figure 5:**
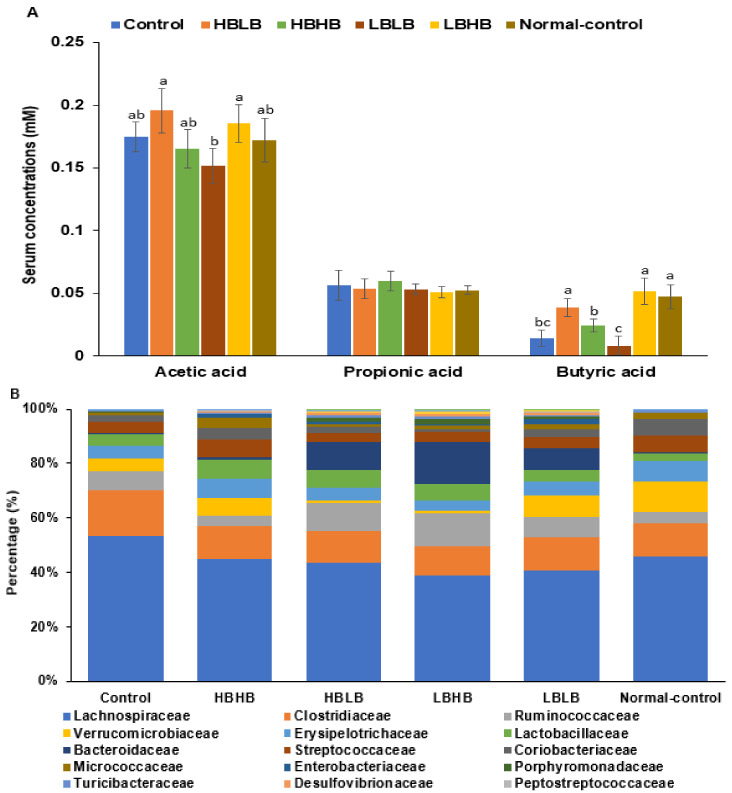

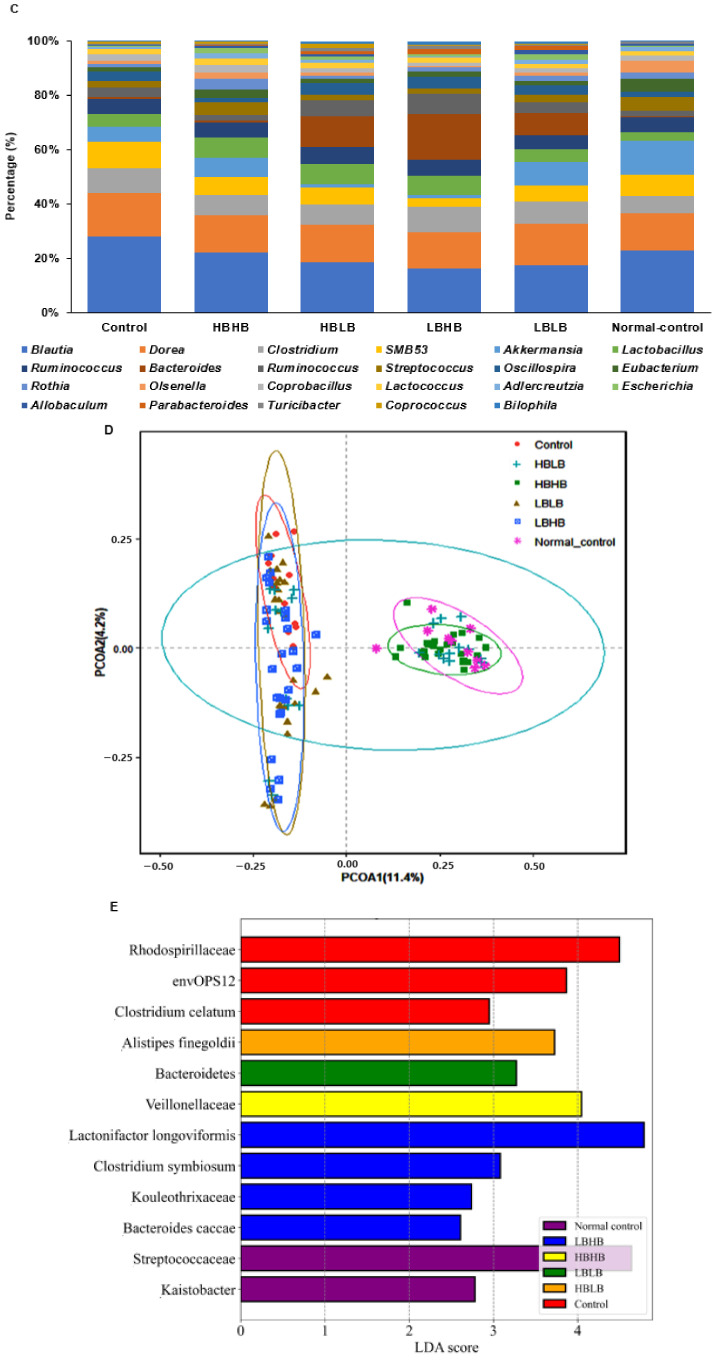

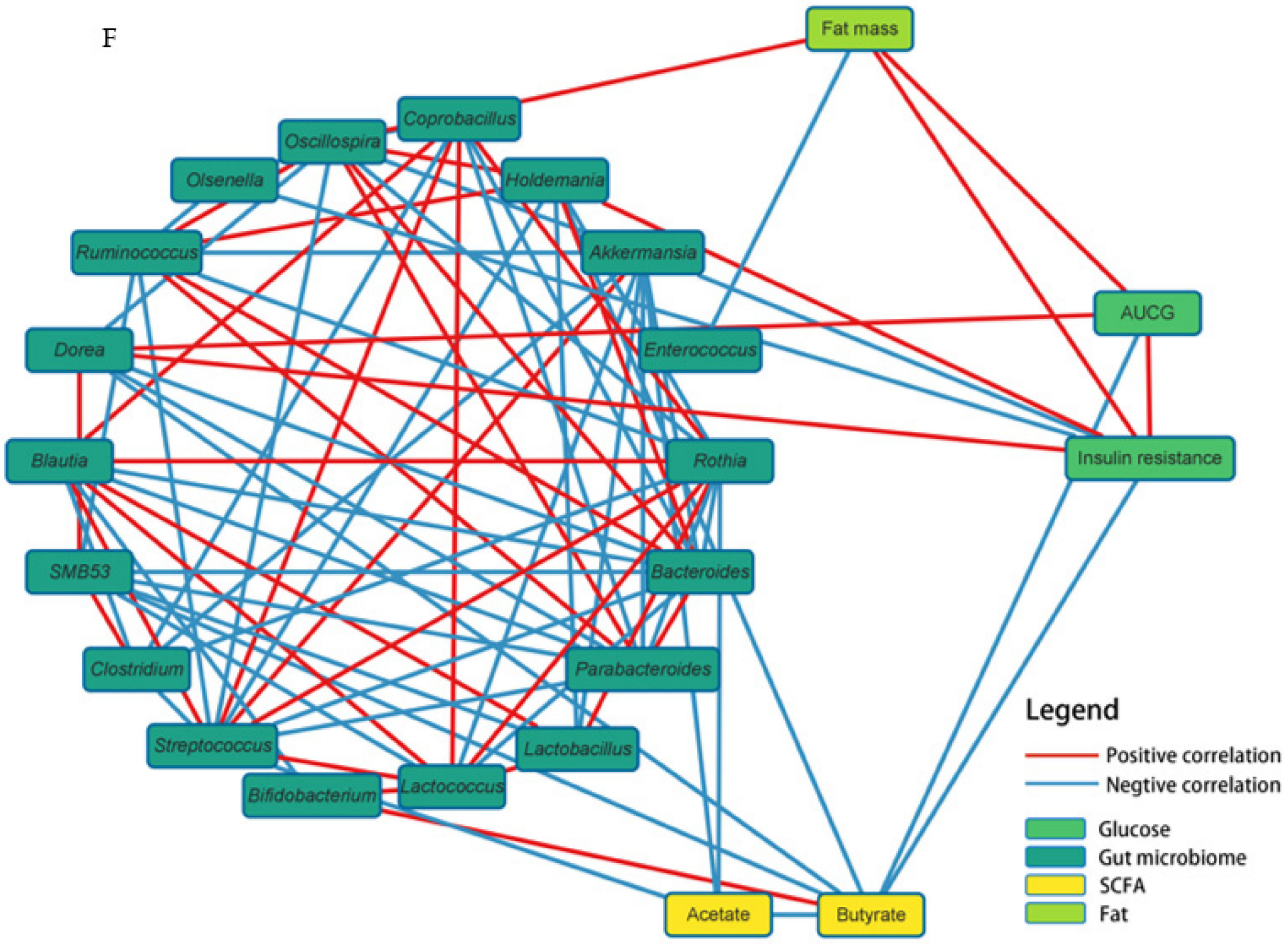
Short-chain fatty acid (SCFA) concentrations and fecal bacteria composition. (**A**). SCFA concentrations in the blood from the portal vein. (**B**). Relative abundance of fecal bacteria from the cecum at the family level. (**C**). Relative abundance of fecal bacteria from the cecum at the genus level. (**D**). β-diversity of fecal bacteria. Colored circles indicated the separation of each group by the assigned color in each group. (**E**). Linear discriminant analysis (LDA) scores in Lefse analysis at the genus level. (**F**). A network analysis of visceral fat, glucose intolerance, insulin resistance, SCFA, and gut microbiota. The correlation coefficient > 0.4 and *p* < 0.0001. SCFA, short-chain fatty acids; AUCG, the area of serum glucose concentration during the oral glucose tolerance test; Fat mass, visceral fat mass. Bars and error bars represent the means ± standard deviations (*n* = 10). HBLB, the rats fed TMK with high contents of *Bacillus* spp. and high biogenic amines; HBHB, the rats fed TMK with high contents of *Bacillus* spp. and low biogenic amines; LBHB, the rats fed TMK with low contents of *Bacillus* spp. and high biogenic amines; LBLB, the rats fed TMK with low contents of *Bacillus* spp. and low biogenic amines. a–c Different letters on the bars indicated a significant difference among the groups by Tukey test at *p* < 0.05.

The acetic acid concentration in the portal vein blood was lower in the LBLB group than in the HBLB and LBHB groups, while the propionic acid concentration was similar in all groups (Figure 5A). Serum butyric acid concentrations were higher in the Normal-control than in the Control, and those in the HBHB and LBHB groups increased similar to the Normal-control (Figure 5A). However, they were lower in the LBLB group than in the other TMK groups (Figure 5A).

### 3.8. Gut Microbiota Community

The gut microbiota composition in the Control was different from that of the Normal-control (Figure 5B). At the family level, the bacteria in Ruminoccocaceae, Clostridiacease, and Streptococcacease increased, but those in Verrucomicroblaceae decreased in the Control group than in the Normal-control group. HBHB and LBHB intake decreased the bacteria in Lachnospiraceae and Clostridiacease and increased those in Verrucomicroblaceae and Lactobacillaceae (Figure 5B). Among TMK types, HBLB and LBHB increased the Bacteriodacease and Ruminoccocaceae levels. At the genus level, *Blautia* and *SMB53* were higher, and *Akkermansia* and *Olsenella* were lower in the Control than the Normal-control (Figure 5C). The HBHB, HBLB, and LBHB groups showed elevated *Akkermansia* and Lactobacillus, while the HBLB, LBHB, and LBLB groups showed an increase in *Bacteroides* and *Ruminococcus* compared to the Control. The changes in bacteria communities suggest that all TMK types increased beneficial bacteria (Figure 5C). At the family and genus levels, the bacterial community of HBHB was similar to the Normal-control.

The Chao and Shannon index showed no significant differences in α-diversity among the groups. The β-diversity showed that PCoA1 and PCoA2 explained the bacterial community diversity by 37.53% and 16.71%, respectively (Figure 5D). The bacterial composition was separated between Control and Normal-control groups in the β-diversity analysis (*p* < 0.001), while HBHB and HBLB groups were separated from the Control group (Figure 5D; *p* < 0.001 in PERMANOVA) while that in HBHB was separately clustered with LBLB and LBHB (*p* < 0.001). The bacterial community in the Normal-control was separated with LBLB and LBHB (*p* < 0.001). On the other hand, it overlapped between the HBHB, HBLB, and Normal-control. Lefse analysis showed that some bacteria were predominant in each group, indicated by linear discriminant analysis (LDA) scores (Figure 5E). The predominant bacteria in the LBHB were *Lactonifactor longoviformis*, *Clostridium symbiosum*, and *Bacteroides caccae*, while the HBHB group contained higher bacteria in Veillonellaceae populations (Figure 5E).

The correlation analysis of fecal bacteria, SCFA, fat mass, and glucose tolerance showed that some gut microbiota was positively and negatively linked to other bacteria. Serum butyrate concentrations were negatively related to serum acetate concentrations and the AUC of serum glucose concentrations during OGTT (*p* < 0.00001; Figure 5F). Serum butyrate concentrations were positively associated with *Bifidobacterium* contents, while they were negatively associated with *SMB53*, *Dorea*, and *Holdemania* contents (*p* < 0.00001). Serum acetate concentrations were negatively related to *Akkermansia* (Figure 5F). Visceral fat mass was positively associated with *Oscillospira* and negatively associated with Enterococcus. The AUC of serum glucose concentrations and insulin resistance were positively associated with visceral fat mass, *Dorea*, and *Holdemania* and negatively linked to *Akkermansia* (*p* < 0.00001; Figure 5F). Therefore, the increment of serum butyrate concentrations mitigated the metabolic disturbance of estrogen-deficient rats, and the relative elevation of *Bifidobacterium* and *Akkermansia* was positively linked to the disturbance.

In metagenome analysis by Picrust2, the HBHB, HBLB, and LBHB groups showed higher levels of aromatic compounds and fatty acid degradation than the Control and Normal-control groups (Table 5). All TMK groups showed increased glutathione metabolism compared to the Control and Normal-control groups (Table 5). Polycyclic aromatic hydrocarbon degradation and unsaturated fatty acid biosynthesis were much higher in the HBLB group than in the Control group (Table 5).

## 4. Discussion

A high salt intake increases the risk of hypertension and metabolic syndrome, particularly in women, elevating the risk of cardiovascular diseases [30]. Furthermore, estrogen deficiency induces metabolic disturbance in energy, glucose, lipid, and bone metabolism [21]. A high salt intake exacerbates metabolic dysfunction [13,31]. Although their relationship remains controversial, a high salt intake induces hypertension in salt-sensitive persons. It has been reported that different salt types influence metabolic disturbances differently [32]. For example, bamboo and solar salts have less hypertensive effects because they contain higher mineral contents, such as potassium and magnesium [32]. Soy sauce usage instead of salt can potentially lessen metabolic disturbances in estrogen-deficient animals. This study examined whether different types of TMK containing low and high biogenic amines and *Bacillus* influenced menopausal symptoms and energy, glucose, and lipid disturbance in OVX rats fed a high-fat diet. This study is novel in finding that TMK with high *Bacillus*, regardless of the content of biogenic amines, alleviated the menopausal symptoms and energy, glucose, and lipid metabolism disturbances in estrogen-deficient rats.

TMK has been made in East Asia counties in somewhat different methods. TMK originated in Korea in 57 BC and is made from meju, fermented soybeans, and salts. Meju and salts (~18 Brix) are added with water and fermented for 40–60 days [33]. The liquid and meju are separated, and the liquids are brewed for 1–2 h. After filtering the liquid, it is aged in the cool and shaded area for over six months, called TMK. During TMK aging, bacteria and their metabolites, including biogenic amines, increase. According to the salt contents and ambient bacteria, the bacteria communities of TMK are different, influencing the TMK quality. In the present study, four different types of TMK were selected according to the contents of *Bacillus* spp. and biogenic amines. Moreover, the biogenic amine contents were higher in TMK with low sodium contents, but the *Bacillus* contents were unaffected by the amounts of sodium. None of the four TMK samples contained aflatoxins. Previous studies have shown that aflatoxin is not detected in different TMK determined by the HPLC method [33]. In TMK, the abundances of fungi are much lower than those of bacteria, but yeasts, such as Debaryomyces and Wickerhamomyces, may be responsible primarily for producing biogenic amines and flavors [34]. Although the fungi community was not determined, the different contents of bioamine indicated the different fungi community in TMK. Interestingly, the predominant bacteria in some TMK (HBHB and HBLB) were *Bacillus subtillus*, similar to doenjang and chungkookjang, but some were not [35]. On the other hand, those in LBHB and LBLB included small portions of the present study. A few studies have been conducted for TMK by NGS: predominant bacteria in TMK are *Staphyloccocus* and *Tetragenococcus*, and TMK contains low *Bacillus* [35]. It suggests that different TMK contains different bacteria communities, and large-scale studies need to be conducted to explore the bacteria community for TMK.

**Figure 6:**
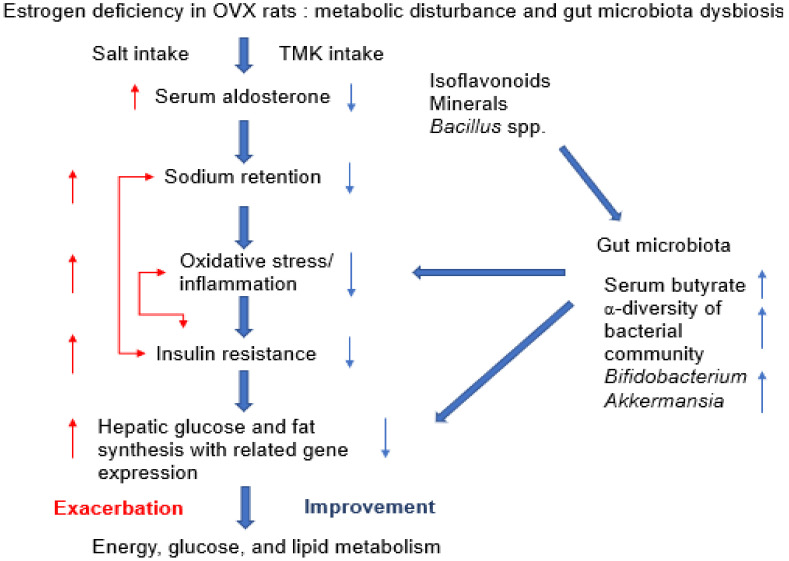
The potential mechanism of traditionally made kanjang (TMK) in the metabolic disturbance in an estrogen-deficient animal model. Salt intake exacerbated metabolic disturbance in estrogen-deficient animals by increasing serum aldosterone concentrations to elevated oxidative stress, inflammation, and insulin resistance, and it disturbed hepatic glucose and fat metabolism. Instead of salt, TMK intake, containing isoflavonoids, minerals, and *Bacillus* spp., improved the disturbance of energy, glucose, and lipid metabolism. TMK reduced serum aldosterone concentrations and increased serum butyrate concentrations by modulating the gut microbiota community, particularly elevating *Bifidobacterium* and *Akkermansia.* TMK reduced oxidative stress, inflammation, and insulin resistance, decreasing hepatic glucose and fat metabolism in estrogen-deficient animals. Red and blue arrows indicated the increase (exacerbation) and decrease (improvement) of each parameter, respectively. Red bidirectional arrows indicated the mutual interaction between the parameters.

Premenopausal women have less risk for metabolic syndrome and cardiovascular diseases, but after menopause, they have an equal or higher prevalence of metabolic syndrome than men [21,36]. It is related to the estrogen loss to no protective activity for metabolic syndrome. Estrogen improves energy, glucose, lipid, bone, and water metabolism to protect against metabolic syndrome risk (Figure 6). Estrogen or phytoestrogen intervention prevented the estrogen-deficient-related symptoms [21,22,29]. On the other hand, a high sodium intake increases the salt sensitivity to hypertension, exacerbating metabolic syndrome, even though women are salt insensitive before menopause [31]. Furthermore, the salt sensitivity to hypertension is inversely correlated with the circulating ovarian hormones [37]. An estrogen deficiency elevates salt sensitivity for blood pressure and exacerbates metabolic disturbance (Figure 6). Salt sensitivity is also associated with increased serum aldosterone concentrations, and it is elevated in people with diabetes, obesity, hypertension, and renal diseases involved in estrogen-deficient symptoms [38]. Furthermore, high salt intake is reported to induce leptin resistance and obesity to increase hepatic fat accumulation in mice [39], and it tended to be linked to nonalcoholic fatty liver disease risk in human beings in a meta-analysis of observational studies [32]. The present study also demonstrated that salt intake increased hepatic triglyceride accumulation by elevating mRNA expression of FAS and SREBP-1c while TMK prevented the increment (Figure 6). Previous studies have shown that different salt sources affect metabolic syndrome because of the contents of other minerals, such as potassium [40,41]. The present study showed that different types of TMK differently reduced serum aldosterone concentrations to alleviate metabolic disturbances and menopausal symptoms by modulating the gut microbiota and increasing SCFA, particularly butyric acid, in an OVX rat, an estrogen-deficient animal. These results indicated that TMK containing higher *Bacillus* spp. had a preventive effect on menopausal symptoms in OVX rats directly by reducing serum aldosterone concentration, oxidative stress, and inflammation and indirectly by modulating gut microbiota (Figure 6).

Fermented foods contain biogenic amines and their safety has been studied. Biogenic amines are organic, basic, nitrogenous compounds. Examples predominant in foods include histamine and tyramine. They are produced mainly by decarboxylation of amino acids in various foods, such as fish and fermented food by *Lactobacillus* and *Bacillus*. They can occasionally accumulate in high concentrations to have adverse effects, such as nausea, headaches, rashes, and blood pressure modulation [42]. Biogenic amines are involved in brain activity, body temperature and stomach pH regulation, gastric acid secretion, immune response, and cell growth and differentiation. The no observed adverse effect level is 2000 ppm for tyramine and 200 ppm for histamine [42]. HBHB and LBHB contained much less histamine and tyramine than the no observed adverse effect level, and they did not affect the metabolism in estrogen-deficient rats in the present study. Furthermore, in the metagenome function of the gut microbiota, all TMK increased the glutathione metabolism and HBHB elevated polycyclic aromatic hydrocarbon degradation compared to the Control. It indicated that some TMK could eliminate the toxic organic compounds and oxidative stress, although they did not show the increment of biogenic amine degradation pathways in the metagenome functions. Further study is needed to investigate TMK to produce the bacteria having biogenic amine degradation pathways.

Fermented soybean products alleviate metabolic disturbance with estrogen deficiency, and their effects are associated with isoflavonoids and the changes in gut microbiota [10,43]. The present study also showed that TMK intake, particularly HBHB and HBLB, modulated fecal microbiota from the cecum and alleviated menopausal symptoms. Although *Bacillus* was the primary bacteria in HBLB and HBHB, it was not elevated in the gut microbiota of the rats in any TMK groups. However, *Bifidobacterium*, *Lactobacillus*, and *Akkermansia* were higher in the HBLB, HBHB, and LBHB groups than in the Control. Previously, chungkookjang fermented with *Bacillus amyloliquefaciens* elevated *Bacillus* in the gut microbiota [44,45]. Therefore, different *Bacillus* spp. may influence the bacteria community differently. TMK of LBLB and LBHB contained different bacteria, but the gut microbiota was partly modulated. Furthermore, the SCFA contents, particularly acetic acid and butyric acid, differed among the groups in the present study. LBLB produced less total SCFA than the other groups, and the butyric acid contents were similar to the Control and less than the other TMK and Normal-control groups. The networking analysis showed that serum butyric acid concentrations were negatively linked to visceral fat mass, glucose intolerance, and insulin resistance and positively associated with *Bifidobacterium* and *Akkermansia* abundance. The improvement of metabolic dysfunction by TMK, especially HBLB and HBHB, was closely linked to the gut microbiota community in estrogen-deficient rats. Additionally, the metagenome function of gut microbiota demonstrated that TMK, especially HBLB and HBHB, stimulated fatty acid degradation and primary bile acid biosynthesis, representing the activation of fat and cholesterol elimination. These results suggested that the modulation of the gut microbiota was associated directly with the bacteria in the TMK, and additionally, metabolites produced by bacteria in the TMK might help modulate the gut microbiota. HBHB had similar gut microbiota to the Normal-control and HBHB, HBLB, and LBHB elevated Lactobacillus in the feces. TMK, particularly HBHB and HBLB intake, improved the gut microbiota to alleviate menopausal symptoms and metabolic disturbance in estrogen-deficient animals.

## 5. Conclusions

Substituting salts with TMK containing high *Bacillus* spp. in the diet showed partial improvement in the menopausal symptoms and metabolic disturbance in estrogen-deficient rats. TMK, regardless of bacteria and biogenic amines, did not have any adverse effects. TMK, particularly with high *Bacillus* levels, instead of salts, was beneficial in alleviating menopausal symptoms by reducing the serum aldosterone concentration and improving the gut microbiota community in menopausal women. A large-scale randomized clinical trial will be needed to validate the effect of TMK on alleviating menopausal symptoms and metabolic disturbance in menopausal women.

## Figures and Tables

**Table 1:** General characteristics of different types of traditionally made kanjang (TMK).

Group	Water (%)	Biogenic Amines(mg/kg)		Na (wt%)	Total Aflatoxin (μg/kg)	Bacteria (%)	
		Histamine	Tyramine			Beneficial	Pathogenic
K-HBLB	66.2 ± 0.55 ^d^	7.60 ± 0.18 ^d^	33.3 ± 0.64 ^d^	8.11 ± 0.18 ^a^	N.D.	79.0 ± 5.14 ^a^	0.023 ± 0.04 ^c^
K-HBHB	72.9 ± 0.96 ^b^	191 ± 3.05 ^b^	1150 ± 0.31 ^a^	5.78 ± 0.10 ^c^	N.D.	72.4 ± 6.12 ^a^	0.001 ± 0.0002 ^d^
K-LBLB	70.5 ± 0.50 ^c^	29.6 ± 0.21 ^c^	155 ± 0.62 ^c^	7.46 ± 0.08 ^b^	N.D.	36.6 ± 1.64 ^b^	0.19 ± 0.02 ^b^
K-LBHB	74.2 ± 0.59 ^a^	214 ± 0.92 ^a^	1009 ± 0.84 ^b^	6.19 ± 0.15 ^c^	N.D.	1.94 ± 1.78 ^c^	33.5 ± 2.74 ^a^

Values represented means ± standard deviation (*n* = 5). K-HBLB, the TMK with high contents of *Bacillus* spp. and high biogenic amines; K-HBHB, TMK with high contents of *Bacillus* spp. and low biogenic amines; K-LBHB, TMK with low contents of *Bacillus* spp. and high biogenic amines; K-LBLB, TMK with low contents of *Bacillus* spp. and low biogenic amines. Wt%, weight percent; N.D., not detected; ^a–^^d^ Different letters on the bars indicated a significant difference among the groups by Tukey test at *p* < 0.05.

**Table 2:** Uterine weight and energy metabolism.

	Control	HBLB	HBHB	LBLB	LBHB	Normal-Control
Uterine weight (g)	0.12 ± 0.01 ^b^	0.12 ± 0.01 ^b^	0.15 ± 0.02 ^b^	0.13 ± 0.01 ^b^	0.16 ± 0.02 ^b^	0.79 ± 0.07 ^a^
Serum 17β-estradiol (pg/mL)	1.42 ± 0.24 ^b^	1.46 ± 0.27 ^b^	1.59 ± 0.21 ^b^	1.43 ± 0.29 ^b^	1.55 ± 0.29 ^b^	6.72 ± 0.98 ^a^
Serum aldosterone (ng/dL)	14.8 ± 2.3 ^d^	22.5 ± 2.8 ^b^	22.9 ± 2.7 ^b^	19.1 ± 2.6 ^c^	21.4 ± 3.2 ^bc^	28.7 ± 3.5 ^a^
Final weight (g)	397 ± 14.0 ^a^	368 ± 8.82 ^b^	349 ± 8.14 ^c^	372 ± 9.97 ^b^	373 ± 11.0 ^b^	316 ± 6.76 ^d^
Body weight gain (g)	240 ± 10.7 ^a^	217 ± 6.99 ^b^	211 ± 7.69 ^c^	240 ± 8.74 ^a^	224 ± 9.12 ^b^	175 ± 6.03 ^d^
Uterine fat (g)	20.1 ± 2.38 ^a^	14.4 ± 2.38 ^c^	14.5 ± 1.10 ^c^	19.0 ± 1.98 ^ab^	19.0 ± 1.71a ^b^	14.0 ± 1.37 ^c^
Retroperitoneal fat (g)	8.22 ± 0.63 ^a^	7.27 ± 0.84 ^b^	6.26 ± 0.65 ^c^	8.55 ± 0.88 ^a^	7.51 ± 0.81 ^b^	5.47 ± 0.48 ^c^
Visceral fat (% of bw)	7.3 ± 0.56 ^a^	5.9 ± 0.53 ^b^	5.9 ± 0.38 ^b^	7.2 ± 0.50 ^a^	7.2 ± 0.55 ^a^	6.1 ± 0.39 ^b^
Food intake (g/day)	14.1 ± 0.93	14.6 ± 1.51	14.4 ± 1.33	15.2 ± 1.19	14.3 ± 0.98	14.3 ± 1.05
Food efficiency (%)	17.1 ± 0.63 ^a^	15.1 ± 0.84 ^b^	14.8 ± 0.59 ^b^	15.8 ± 0.58 ^b^	16.6 ± 0.71 ^ab^	13.2 ± 0.46 ^c^

Values represented means ± standard deviation (*n* = 10). Food efficiency = food intake/body weight × 100. HBLB, the rats fed TMK with high contents of *Bacillus* spp. and high biogenic amines; HBHB, the rats fed TMK with high contents of *Bacillus* spp. and low biogenic amines; LBHB, the rats fed TMK with low contents of *Bacillus* spp. and high biogenic amines; LBLB, the rats fed TMK with low contents of *Bacillus* spp. and low biogenic amines. Bw, body weight. ^a–^^d^ Different letters in each variable indicate significant differences in one-way ANOVA at *p* < 0.05.

**Table 3:** Glucose metabolism and lipid profiles.

	Control	HBLB	HBHB	LBLB	LBHB	Normal-Control
Serum glucose (mg/dL)	141 ± 6.51 ^a^	130 ± 7.20 ^b^	132 ± 3.17 ^b^	143 ± 5.61 ^a^	137 ± 7.02 ^ab^	113 ± 3.57 ^c^
Serum insulin (mg/dL)	2.04 ± 0.31 ^a^	1.10 ± 0.30 ^b^	1.25 ± 0.31 ^b^	2.10 ± 0.37 ^a^	1.51 ± 0.38 ^ab^	0.99 ± 0.16 ^b^
HOMA-IR	12.8 ± 1.12 ^a^	6.31 ± 0.67 ^c^	7.11 ± 0.55 ^c^	13.4 ± 0.10 ^a^	9.27 ± 0.90 ^b^	4.97 ± 0.30 ^d^
Serum total chol (mg/dL)	123 ± 7.29 ^a^	116 ± 4.69 ^a^	115 ± 5.62 ^b^	102 ± 9.28 ^c^	103 ± 4.62 ^c^	118 ± 5.67 ^a^
Serum HDL (mg/dL)	28.8 ± 2.08 ^d^	56.3 ± 5.46 ^b^	48.3 ± 2.47 ^c^	34.8 ± 3.64 ^c^	37.3 ± 2.21 ^c^	68.4 ± 5.07 ^a^
Serum TG (mg/dL)	73.7 ± 4.36 ^a^	70.2 ± 4.12 ^a^	62.9 ± 2.8 ^b^	70.6 ± 6.04 ^ab^	64.8 ± 2.6 ^b^	54.3 ± 3.04 ^c^
Serum LDL (mg/dL)	79.6 ± 6.27 ^a^	43.0 ± 3.66 ^c^	54.3 ± 5.26 ^b^	53.0 ± 3.39 ^b^	51.6 ± 3.92 ^b^	38.6 ± 3.97 ^d^
Serum TNF-α (pg/mL)	56.8 ± 3.9 ^a^	46.1 ± 3.7 ^c^	50.3 ± 3.6 ^b^	52.5 ± 4.2 ^ab^	51.4 ± 4.1 ^b^	44.4 ± 3.8 ^c^
Serum malondiladhyde (μM)	43.8 ± 3.8 ^a^	38.8 ± 3.4 ^b^	39.5 ± 3.5 ^b^	40.4 ± 3.3 ^ab^	41.3 ± 3.8 ^ab^	34.5 ± 3.6 ^c^

Values represented means ± standard deviation (*n* = 10). HBLB, the rats fed TMK with high contents of *Bacillus* spp. and high biogenic amines; HBHB, the rats fed TMK with high contents of *Bacillus* spp. and low biogenic amines; LBHB, the rats fed TMK with low contents of *Bacillus* spp. and high biogenic amines; LBLB, the rats fed TMK with low contents of *Bacillus* spp. and low biogenic amines. HOMA-IR, homeostatic model assessment for insulin resistance; HDL, high-density lipoprotein; TG, triglyceride; LDL, low-density lipoprotein; TNF-α, tumor-necrosis factor-α. ^a–^^d^ Different letters in each variable indicate significant differences in one-way ANOVA at *p* < 0.05.

**Table 4:** Liver damage index and hepatic glucose and lipid metabolism.

	Control	HBLB	HBHB	LBLB	LBHB	Normal-Control
Serum AST (IU/L)	51.9 ± 5.32 ^a^	34.1 ± 4.17 ^b^	28.3 ± 7.22 ^b^	21.0 ± 5.77 ^c^	29.7 ± 8.88 ^b^	20.0 ± 1.66 ^c^
Serum ALT (IU/L)	36.6 ± 6.54 ^a^	26.3 ± 2.99 ^b^	15.4 ± 1.82 ^d^	22.8 ± 2.00 ^c^	20.1 ± 2.41 ^c^	18.3 ± 2.66 ^d^
Liver glycogen (mg/g tissue)	68.0 ± 6.90 ^c^	75.2 ± 7.20 ^b^	85.6 ± 9.0 ^a^	68.5 ± 3.71 ^c^	81.9 ± 4.42 ^a^	84.5 ± 3.93 ^a^
Liver triglyceride (mg/g tissue)	45.6 ± 6.24 ^a^	16.7 ± 3.18 ^d^	18.8 ± 2.66 ^d^	23.2 ± 3.50 ^c^	28.6 ± 8.12 ^b^	13.0 ± 3.40 ^d^
Liver relative mRNA of PPAR-γ (AU)	1 ^c^	2.45 ± 0.45 ^a^	1.99 ± 0.43 ^b^	1.21 ± 0.34 ^c^	0.89 ± 0.15 ^c^	2.53 ± 0.49 ^a^
Liver relative mRNA of FAS (AU)	1 ^a^	0.43 ± 0.09 ^c^	0.60 ± 0.16 ^b^	0.66 ± 0.14 ^b^	0.93 ± 0.17 ^a^	0.37 ± 0.08 ^c^
Liver relative mRNA of SREBP-1c (AU)	1	0.94 ± 0.18	0.99 ± 0.17	0.89 ± 0.14	0.91 ± 0.15	0.94 ± 0.17

Values represented means ± standard deviation (*n* = 10). HBLB, the rats fed TMK with high contents of *Bacillus* spp. and high biogenic amines; HBHB, the rats fed TMK with high contents of *Bacillus* spp. and low biogenic amines; LBHB, the rats fed TMK with low contents of *Bacillus* spp. and high biogenic amines; LBLB, the rats fed TMK with low contents of *Bacillus* spp. and low biogenic amines. TG, triglyceride; AST, aspartate aminotransferase; ALT, alanine aminotransferase; PPAR-γ, peroxisome proliferator-activated receptor-gamma; AU, arbitrary unit; FAS, fatty acid synthase; SREBP-1c, sterol regulatory element-binding protein-1c. ^a–^^d^ Different letters in each variable indicate significant differences in one-way ANOVA at *p* < 0.05.

**Table 5:** Metagenome function of the fecal bacteria from the cecum.

	Control	HBLB	HBHB	LBLB	LBHB	Normal-Control
Fatty acid degradation	0.252 ± 0.003 ^c^	0.270 ± 0.003 ^b^	0.285 ± 0.004 ^a^	0.273 ± 0.004 ^a^	0.280 ± 0.003 ^a^	0.260 ± 0.005 ^c^
Primary bile acid biosynthesis	0.0306 ± 0.0019 ^b^	0.0439 ± 0.002 ^a^	0.0346 ± 0.002 ^a^	0.0439 ± 0.004 ^a^	0.0461 ± 0.004 ^a^	0.0275 ± 0.001 ^b^
Biosynthesis of unsaturated fatty acids	0.003 ± 0.004 ^b^	0.003 ± 0.008 ^b^	0.010 ± 0.001 ^a^	0.007 ± 0.001 ^ab^	0.003 ± 0.001 ^b^	0.007 ± 0.001 ^ab^
cAMP signaling pathway	0.008 ± 0.0002 ^bc^	0.0007 ± 0.0001 ^c^	0.003 ± 0.0003 ^a^	0.001 ± 0.0002 ^b^	0.001 ± 0.0002 ^b^	0.002 ± 0.0004 ^ab^
Glutathione metabolism	0.2525 ± 0.005 ^b^	0.3031 ± 0.006 ^a^	0.291 ± 0.006 ^a^	0.293 ± 0.008 ^a^	0.3118 ± 0.005 ^a^	0.2574 ± 0.005 ^b^
Polycyclic aromatic hydrocarbon degradation	0.0015 ± 0.0003 ^c^	0.0014 ± 0.0003 ^c^	0.0059 ± 0.0005 ^a^	0.0028 ± 0.0004 ^b^	0.0021 ± 0.0005 ^b^	0.0038 ± 0.0001 ^a^

Values represent the means ± standard deviations (*n* = 10). HBLB, the rats fed TMK with high contents of *Bacillus* spp. and high biogenic amines; HBHB, the rats fed TMK with high contents of *Bacillus* spp. and low biogenic amines; LBHB, the rats fed TMK with low contents of *Bacillus* spp. and high biogenic amines; LBLB, the rats fed TMK with low contents of *Bacillus* spp. and low biogenic amines. ^a–c^ Different letters on the bars indicated a significant difference among the groups by Tukey test at *p* < 0.05.

## Data Availability

The data presented in this study are available on request from the corresponding author.
